# The Use of Research Findings on Self-Regulated Learning by Teachers and Students in an Australian High School

**DOI:** 10.3390/bs15121644

**Published:** 2025-11-30

**Authors:** Michael J. Lawson, Stella Vosniadou, Helen Stephenson, Lachlan McFarlane, Jason Loke, Tracy Robinson, Ben Cullen, Jess Rogers, Stew Nancarrow, Brenna Andrews, Nathan General, Twinkle Gomes, Sophie Calliss, Paige Harrison

**Affiliations:** 1 College of Education, Psychology and Social Work, Flinders University, Adelaide, SA 5042, Australia; 2 Education Futures, University of South Australia, Adelaide, SA 5000, Australia; helen.stephenson@unisa.edu.au; 3 Blackwood High School, Blackwood, SA 5051, Australiastew.nancarrow869@schools.sa.edu.au (S.N.);

**Keywords:** self-regulated learning, teacher use of research findings, student promotion of self-regulated learning

## Abstract

Reviews of the use of research ideas by teachers indicate that such use is less frequent than it could be. One area in which such a pattern of use is apparent concerns the effective promotion by teachers of strategies for the self-regulation of learning (SRL). Despite evidence of the importance of explicit SRL promotion, it is apparent that findings from research are not being used by many teachers. This report provides details of an instance of effective use of research findings by both teachers and students in one Australian secondary school following the involvement of a group of teachers from the school in a professional development program related to SRL and to the ICAP framework for cognitive engagement The report provides details of teacher-designed classroom interventions and activities of a student group which included use of the research findings with other students. From the research, two factors seen to have stimulated use of the research ideas emerge. The first is the contiguity of the professional development program and the actions of school leaders, teachers, and students. The second factor is the continuing involvement of researchers with teachers. The report provides an example of how students can engage in promotion of SRL strategies to other students.

## 1. Introduction

The ability to manage and control one’s learning has become an important requirement for navigating today’s complex and digitized world (Bjork et al., 2013). Usher and Schunk (2018) describe SRL as “The process of systematically organizing one’s thoughts, feelings, and actions to attain one’s goals” (p. 19), and it is described in several theoretical models that overlap in significant detail (e.g., Boekarts, 1997; Efklides, 2017; Mayer, 2017; Pintrich, 1999; Schunk & Zimmerman, 2013; P. H. Winne, 2001). Current research in this area provides strong evidence of the benefit of SRL strategy promotion and of procedures that can help students acquire such strategies (e.g., Dunlosky et al., 2013; Fong et al., 2023; MacArthur, 2012; Schunk & Greene, 2018; Sitzman & Ely, 2011; P. Winne, 2018). However, other research findings show that the explicit promotion of such learning strategies in lessons is not as widespread as might be expected (Dignath & Mevarech, 2021; Dignath & Veenman, 2021; Vosniadou et al., 2024). This suggests that findings from research on SRL strategies are not being used in many lessons where they could be of benefit.

Consideration of what influences the use of educational research findings in schools has been of concern for some time (e.g., Buchmann, 1984; Hemsley-Brown & Sharp, 2004; Griffin & Barnes, 1986) and is still a significant contemporary concern (e.g., Gleeson et al., 2024; Joram et al., 2020; Mausethagen et al., 2025; Penuel et al., 2017; Sato & Loewen, 2022). In their recent review of the field, Mausethagen et al. (2025) argue that, despite an increasing body of research on this topic, research use by teachers is still quite limited. This situation is of concern for educational researchers generally, and it is also of concern to those of us who carry out research on SRL and its promotion by teachers in classroom lessons.

The case report presented here provides an example where the research use by teachers emerged from the teachers’ involvement in an experimental Professional Development Project (PDP) on the indirect and direct promotion of self-regulated learning (SRL) in the classroom. The results discussed here were derived from instances of action research carried out by teachers and from data provided in interviews with school leaders, teachers, and students. We suggest that this report points to how researchers, teachers, and students can effectively use and extend findings from research that address the problem of promotion of SRL. We see that procedures initiated and adopted in the research project, and the practices established by teachers and students following the research, provide useful ideas for teachers, students, and researchers about how they might exploit important findings from contemporary educational research in classroom lessons and private study. The report provides details of the participants and procedures involved in the PDP, and then sets out the program of application of ideas from the PDP within the school.

In the sections that follow, we first describe the PDP, then continue with a description of the school in which the case study took place, and finish with a summary of the three components of the school program on SRL promotion.

### 1.1. Professional Development Program

The PDP designed for the research project centered on two issues of current SRL research concern. The first of these was the direct promotion of SRL knowledge and strategies, focusing on the knowledge that students need to manage their learning effectively, involving cognitive, metacognitive, motivational, and affective components of learning. The second involved the indirect promotion of SRL. The Interactive, Constructive, Active, Passive (ICAP) theoretical framework developed by Chi and Wylie (2014) was used for said indirect promotion (see Vosniadou et al., 2025). In 2020, participants from the school studied materials that focused on the nature of SRL, on specific SRL capabilities, and on how SRL strategies could be directly promoted in classroom lessons. In the consideration of these topics, participating teachers examined videos of lessons, including videos of their own teaching, to identify both SRL strategies and instances of promotion of these strategies. In addition, the teachers also coded transcripts of lessons to identify different SRL capabilities and different types of SRL promotion.

The second issue in the PDP in 2020 was indirect promotion of SRL, focusing on the levels of cognitive engagement in tasks presented by teachers to their students, in the context of the ICAP cognitive engagement framework of Chi and Wylie (2014). Here, the focus was on the nature of cognitive engagement in relation to the four modes of cognitive engagement identified in the ICAP framework: Interactive, Constructive, Active, and Passive cognitive engagement. The ICAP framework also includes specification of the different knowledge change processes associated with each of these four modes. Research investigating the effects of training teachers in use of the ICAP framework shows that student learning is more effective when students are engaged in Interactive or Constructive activities than when they are engaged in Passive or Active activities (Chi & Boucher, 2023).

The participating teachers undertook activities that involved coding of lesson tasks for ICAP levels and were also required to identify how specific lesson tasks could be changed to increase levels of cognitive engagement. An important feature of the PDP was that the SRL and ICAP theoretical frameworks were presented in the one PDP so that the ideas central to each framework were brought into interaction. One example of this is the way that the design of tasks being considered with the ICAP framework was seen to influence the type of SRL promotion that could be generated by the teacher. As an example of this, a task in a mathematics lesson discussed in the PDP involved the use of a self-explanation strategy but also required students to engage in Interactive cognitive engagement about ways to use the strategy.

### 1.2. The School in Which the Program Took Place

Blackwood High School is a co-educational government secondary school located in suburban Adelaide with an enrolment of around 1200 students. In 2024, the school had 80 teaching staff and an Index of Community Socio-Educational Advantage (ICSEA) score of 1069; the average ICSEA score for Australian schools is 1000. Twelve percent of students had language backgrounds other than English, and one percent had an Indigenous Australian background. Students in the school study the Australian Curriculum across Years 7 to 10 and the South Australian Certificate of Education (SACE) in Years 11 and 12. Seven experienced staff from the school accepted the invitation to participate in the PDP at a local university.

The state-wide education system policy environment for the school has, at its core, four stated areas of impact: wellbeing, learner agency, effective learners, and equity and excellence (Department for Education, South Australia, n.d.). This system-wide policy places students as partners in their learning who need to have the capacity to cognitively and confidently engage in their learning, know how to learn in any given context, and know how to manage and impact their learning experiences—empowering all learners to thrive and prosper. It was in this policy context that, in 2020, staff from the school undertook the research PDP and subsequently developed their own teaching strategy promotion initiatives and worked on these initiatives with students.

## 2. Materials and Methods

### 2.1. Participants

Seven teachers from the school were participants in the PDP. These teachers all had greater than five years of teaching experience. After the completion of the PDP, participants were invited by the researchers to consider the possibility of interventions that they could undertake in their own classrooms. Subsequently, in 2021, five teachers, including Leader 2, planned such interventions and discussed their design in meetings with the research team. Teachers in the areas of Humanities, Social Science, Technology, and Mathematics undertook and reported on these interventions. Three of the five teachers also participated in interviews at the end of 2023.

In 2022, a further set of teacher-designed learning strategy interventions, referred to as ‘Learning Sprints’, were delivered, with a focus on strategies for senior-years classes, Years 11 and 12. In gathering data for this report, one of the teachers participating in the Learning Sprint program was interviewed at the end of 2023 (Teacher F). Teacher F worked with the Innovative Pedagogy Leader and other teachers to plan an intervention that focused on use of a retrieval practice strategy for students in her Year 12 Biology class.

Interviews were also conducted at the end of 2023 with four students who had been leaders of the Learning Culture Group (LCG). Three of the students interviewed were current leaders of the LCG, and the fourth student was, at the time of the interview, a university student who had been a leader of the LCG in the year prior to university entry.

### 2.2. Procedure

#### 2.2.1. School Program of Use of Research Ideas

The school program involving use of research ideas from the PDP considered in this case report involved three components.

#### 2.2.2. Teacher-Designed Interventions

In 2021, the first part of this program involved a set of action research interventions (Cohen et al., 2017) by teachers who had been involved in the research. Interventions varied in length, with most being about two to three weeks in duration, although the Technology intervention ran for five weeks. Across this time, the interventions were enacted in each lesson. Following on from the themes discussed in the PDP resources, the teachers combined the use of constructive and interactive lesson tasks with the explicit promotion of learning strategies (Dignath & Veenman, 2021). Teachers were advised to model use of the strategies during the lesson and to help students to identify strategies by name, as well as to provide students with periods of practice in strategy use during several lessons.

#### 2.2.3. Learning Sprints

In the second component of the school program, enacted in 2022, leaders in the School decided to initiate further learning strategy interventions and to directly promote the use of learning strategies in all classes involving students in Year 12, the final year of schooling. This program of professional learning initiated and carried out by school staff was identified as the Learning Sprint program. It is through completion of studies in Year 12 that students qualify for the certificate recognizing successful completion of secondary education, the SACE. The final stage of the year includes external assessments and/or examinations for all students intending to fulfill the SACE requirements. Participating teachers met with two other teachers in continuing group discussions prior to choosing a strategy and procedure for strategy promotion that they would use in their class. This discussion also required teachers to prepare an assessment process for evaluating the effects of strategy promotion. The strategies were explicitly promoted to students through detailed description and explicit modeling, as well as repeated use and discussion in the lesson series. Later that year, the teachers who had designed and carried out Learning Sprints presented details of the Learning Sprint strategy programs and their effects to other teachers in the School and in other schools, as well as to research team members. In 2023, the learning strategy program was extended to other year levels across the school.

Support from the school included release time to prepare materials and discussion of the possibilities for design of sprints in staff meetings. Teachers chose one strategy from several that had been introduced and designed an intervention to implement it. The strategies were retrieval practice, interleaved practice, spaced practice, elaboration, activating students as owners of their own learning, and activating peers as resources for one another (see Dunlosky et al., 2013; Gurung & Dunlosky, 2023; Willingham, 2023). Teachers completed the six-week sprint, collating evidence of impact to share in a final report that formed the basis for the presentation to colleagues, researchers, and teachers from nearby schools. The measures used to gather information about the effects of the sprint included comparison of student work at the start and end of the sprint, student reflections and survey data, formative work, and summative assessment results.

#### 2.2.4. The Learning Culture Group

The final part of the school program discussed here is the nature and activity of the LCG, a student group that focused on the promotion of SRL knowledge and strategies to other students. As part of their consideration of the learning environment in the school, in the year prior to participation in the PDP, school leaders established a student LCG. The purpose of the LCG was to work with school leaders, teachers, and students on ways to improve the environment for learning in the school and improve students’ knowledge about learning. Prior to this, student leadership had focused primarily on wellbeing activities and events, with less focus on student leadership with respect to learning within the classroom. Members of the LCG were not necessarily student leaders but students who were interested in the topic of learning and the ways that knowledge about learning could be further developed by students. The established group met every two weeks under the supervision of Leader 2 and developed a program of discussions, sought out resources, such as those provided by the Learning Scientists website (Learning Scientists, 2025), and invited attendance by other students concerned about their learning. They also met with teachers and school leaders on student-free days and at formal meeting times to promote pedagogical initiatives and contribute to the school curriculum and learning design. At the end of 2022, the students involved in the LCG also designed and presented seminars on learning strategies specific to students who would enter Year 11 in 2023.

### 2.3. Measures

#### 2.3.1. Teacher-Designed Intervention Measures

The data considered in this report is derived from two sources. Written reports of teachers who had delivered the SRL strategy promotion intervention to their classes served as a source. Toward the end of 2021, these teachers presented reports on the results of the interventions and discussed these results with members of the research team. These reports provided details of the objectives of the intervention, details of the activities undertaken in the intervention, and details of the use of measures used to evaluate the effects of the interventions, as shown in Table 1 and Table 2. Subsequently, in 2021, these teachers presented the results of these interventions to their colleagues during a student-free day of professional learning for teachers. This day also included a presentation by students in the LCG to teachers and researchers that involved the use of the ICAP framework to illustrate differences between lesson tasks that would stimulate either Passive or Constructive and Interactive levels of student cognitive engagement.

#### 2.3.2. Interviews

The second source of data was interviews with two school leaders, three teachers, and four students, conducted at the end of the 2023 school year. The interviews ran between 45 min and 1 h in length. The leaders were one of the school’s executive leadership team (Leader 1) and a Mathematics teacher who was also the Innovative Pedagogy Leader for the school (Leader 2). The responsibilities of Leader 2 involved consideration of approaches taken to teaching and learning within the school, in order to develop projects that would facilitate desired change in these approaches and to coordinate the student LCG. The other teachers, who all had more than five years of teaching experience, taught in the areas of Humanities, Biology, and Technology.

For all of the leader and teacher interviews, questions addressed the following topics: (1) reasons for participating in the PDP and in the subsequent school program that used research ideas; (2) the effects of their involvement on their teaching or learning and on the school, including the use of specific research ideas included in the PDP; (3) what use they had made of the research ideas; (4) whether they would recommend use of the research ideas to other teachers or students; and (5) any advantages or disadvantages associated with the use of the research ideas. The questions addressed to students focused on the following issues: (1) the nature and use of the strategies they had learned; (2) the extent of their use of the strategies, including in other subject areas; (3) the effects of the use of the strategies; and (4) whether they would recommend any specific strategies to other students. The analysis of the interviews focused on the following themes: (1) teachers’ reasons for involvement in research and the use of research ideas; (2) teachers’ and students’ knowledge and methods of use of research ideas; and (3) the effects of use of the research ideas on teaching or learning and whether they would recommend that other teachers or students use the research ideas in the ways used in this school.

The interview protocol was approved by the university ethics committee and the Government Department for Education responsible for the school, and informed consent was obtained from all participants who volunteered to take part in the research. Data from the interviews is used in the following discussion of the teacher and student activities that used ideas from the PDP. The interview questions are available from the authors.

## 3. Results and Discussion

### 3.1. The PDP and Use of Research Ideas

The detailed effects of this professional development program have been examined and reported on by the research team (Vosniadou et al., 2023, 2024). However, in the context of this paper, it is also relevant to consider features of the PDP that could be seen to have impacted on the subsequent use of research findings by the teachers from this school.

One of the major obstacles to use of research identified by Sato and Loewen (2022) was the separation of the worlds of researchers and teachers such that research was seen by teachers as not addressing the practical realities of classroom teaching. As noted above, both the SRL and ICAP components of the PDP involved teachers critically considering ways to further promote SRL strategies in lessons and ways to redesign lesson tasks to increase levels of cognitive engagement. As part of this element of the PDP, teachers, at times, were undertaking this critical analysis using videos of their own teaching, of their own strategy promotion actions, and of their own lesson task design. These elements of the PDP can be seen to provide opportunities for the program to reduce some of the practical obstacles identified by Sato and Loewen (2022) that make use of research ideas more difficult. In this respect, it is relevant to note that the research discussed here was not what Mausethagen et al. (2025) refer to as “externally produced research” (p. 23). The fact that these teacher participants were engaged in critical examination of their own practice during the research can be seen to be different in nature to research where researchers might ask teachers to use research ideas that had been generated in a research program where the teachers in this school were not involved. In this sense, elements of the PDP experienced by the teachers in this school can be seen to have made more apparent the direct practical relevance of the research ideas to participants’ teaching practices.

The interviews with participants suggested that they did see possibilities for further use of ideas in the PDP, uses that could stimulate change in the broad learning environment of the school. These changes were also seen to be compatible with practical action in areas like student agency that were part of the recent school system policy related to learning. A key idea for Leader 1 was the focus of the PDP on “building effective learners and giving learners the language to speak about their learning.” For Teacher E, the focus of the PDP addressed an issue that had arisen in her teaching: “I felt that, as teachers, we always seem to assume that students know how to learn, that somehow they just pick up strategies to learn.”

The state of student knowledge about how to learn, and especially how to learn effectively in their private study periods, was of particular concern. Discussions among teachers and emerging from in-school surveying of students about the effectiveness of their independent study time suggested that change was needed to further develop their knowledge of how to learn effectively during these periods.

“We surveyed them, we talked to them [in] focus groups, and also [had] the students reflecting on their study lines [free study periods]. You know, how effectively do they think they use study lines for other students, their peers, what they observe. And quite widely, the feedback was the study lines aren’t much use at all. Most students don’t make any use of them”.

(Leader 1)

Discussions with teachers and students suggested to Leader 1 that

“We haven’t actually helped the students learn how to study…And so when they came to that independence in that study line, they didn’t know how to grapple with the challenge of doing something where they didn’t have a teacher to provide that explicit scaffold or instruction to help them become unstuck.”

School leaders were also aware that many students achieved lower grades in the external assessments associated with Year 12 than they did in their school-based assessments. The possibility was then considered that such a pattern of performance could be associated with students’ lack of knowledge of how to address an issue like activating knowledge acquired much earlier in the school year. Leader 1 noted, “And so is it then a challenge of that recall capacity. And are they not actually studying and strengthening that ability to recall that information when it’s needed for that exam?” Views such as these, expressed in interviews, suggested that the research ideas included in the PDP were seen to be relevant to the needs of teachers and students in the school.

### 3.2. Results of the Teacher-Designed Interventions

The topics and details of the procedures used in each teacher’s intervention are shown in Table 1. For Teacher E, involvement in the PDP caused her to reflect on her students’ knowledge about controlling their learning:

“I realized that not everybody in my classroom had the same access to understanding around learning. I felt I was a good teacher, but I felt like I wasn’t explicitly teaching my kids how to learn…I wanted to see if, by explicitly teaching these strategies, or pointing them out when they were being used [by students], [that] would improve learning outcomes.”

Part of that explicit discussion about learning was what this teacher referred to as ‘calling out’ the examples of active learning, giving explicit recognition during a class to student behaviors that were examples of effective learning strategy use and of the Constructive and Interactive engagement discussed in the ICAP framework. This ‘calling out’ could be seen as a procedure that could address an issue arising in ICAP research (Chi &Boucher, 2023), where students either do not apply an available learning process or “downgrade” the level of cognitive engagement of their learning actions.

In the Mathematics intervention, Teachers A and B used the ICAP framework to design Interactive engagement tasks and required students to use a self-explanation strategy (Renkl & Eitel, 2019). As part of their Algebra equation problem solving, students worked through a “Convince Yourself, a Friend, a Skeptic” interactive procedure that required them to

- Explain problem-solving steps to self;
- Explain what you know, and how you know this is so, to a friend who provides feedback, include any words, pictures, or numbers. Identify strategies used this week;
- Explain why what you know is true and provide evidence; address counterarguments, presenting explanation to a skeptical peer who will require additional evidence and provides feedback. Include any words, pictures, or numbers.

In the Technology classes, Teacher C observed that, as he incorporated the ICAP intervention approaches into his product-design classes, he observed more

“working together, planning tasks in the workshop, and then helping each other, even if it’s just they’re doing the same task, and they can take photos for each other and share them and talk about how do they come up with a better way to do this project.”

**Table 1:** Teacher-designed class interventions (2021).

Year 8 Algebra (Teachers A and B)	Year 8 Technology (Teacher C)	Year 8 Civics and Citizenship (Teacher D)	Year 9 Humanities (Teacher E)
Explicit teaching by teacher of SRL self-explanation strategy steps in problem solving involving naming, teacher modeling and class practice in use of the strategy steps in Year 8 Algebra. Changed design of tasks to make these more interactive, to enable students to collaboratively explain thinking and provide justifications for explanations related to problem-solving steps. Students explained steps to themselves, then to a friend, then to a skeptical peer. Visual display of explanation steps on board. Students develop portfolio of explanations and slides including photos of their best explanations.	Explicit naming and modeling of use of product testing and evaluation processes by teacher. Use of tasks stimulating Interactive cognitive engagement by students in small groups. In these groups, students used templates requiring Interactive engagement during their evaluations of products they had constructed in class technology exercises. Small-group discussion required critique of own and other students’ products prior to final evaluations.	Explicit teaching and modeling of strategies for text comprehension, using strategies for annotation and summarizing. Provision of templates for annotation and summarizing procedures. Students worked in groups through Interactive cognitive engagement tasks requiring description of their own annotations and summaries, and then justified these to another student and then swapped roles to provide critiques and require justification of the peer’s annotations and summaries.	Explicit teacher description, explanation, and modeling of strategies involving question posing and answering for notetaking, mind-mapping, and post-task reflection. Think–Pair–Share activities involving strategy descriptions, explanation with peer, and then with the class; feedback on strategies provided by other students. For post-task reflections, students used prompts: What was the task? How did I do it? How successful was I? What can I change in future tasks? Diary prompts and use of journals: Statements of lesson intentions and structure; Application of success criteria; Noting of possible learning strategies for the lesson. Classroom posters displayed steps of strategies.

These effects are consistent with findings in other ICAP research discussed by Chi and Boucher (2023). Teacher C also noted an impact of this change, in terms of students’ mode of engagement, on class management.

“In the workshop, there’s maybe 20 kids, but they’re not friends. They don’t hang out outside of class, but [in this Interactive mode] they get to the point where they can work together well and actually go to each other and ask for advice or help with something.”

In the classes of Teacher D, the change in student behavior in relation to the learning strategies was characterized by a gradual increase in student responsibility for initiation of strategy use in text comprehension exercises. The changes in student performance noted by Teacher D are consistent with other reading comprehension interventions involving explicit strategy promotion (e.g., Huang & Yang, 2015).

As indicated in Table 1, Teacher E also provided students with posters that were displayed in the classroom to serve as reminders of the steps involved in a certain strategy. For this teacher, a key issue was helping students to realize that they had real agency in their learning: “Getting them to know that it is them doing the thinking, not me.” To provide a ‘hook’ for this idea, and to make explicit the link between their brain action and learning, for some part of the intervention, Teacher E referred to students as her ‘neuro babies’, using that expression as shorthand for important mental connections between ideas or concepts: “And the kids love it whenever they do something, and I see that they’ve made that connection, I go ‘Neuron everybody, Neuron baby, we’ve just had one’ and they get really excited about it.” The teacher’s actions here provide an example of making the learning actions visible to other students (Hattie, 2012). The effects of the intervention on question posing noted by this teacher have also been reported in related research (e.g., Kaberman & Dori, 2009).

In keeping with the explicit strategy promotion discussed during the PDP, teachers in these interventions also provided students with information during lessons on why the strategies helped the learning process, so that students might further develop their general knowledge of how learning could occur. In a sense, through these procedures, the teachers were providing students with both the theoretical rationale for the strategy and explicit promotion of the strategy and practice in its use. As shown in Table 1 and Table 2, the teachers gathered detailed information that would enable judgements to be made about the effects of the strategy interventions. These measures included grades on class exercises and assignments, student self-reports on strategy use, and scoring on measures such as the International Baccalaureate Middle Years Program (IBMYP) criteria for grading student performance (see Queensland Academy for Science, Mathematics and Technology, 2021).

Information on the effects of the teacher-led interventions identified by the teachers is shown in the lower section of Table 2. As noted earlier, it must be recognized that these effects did not arise from true experimental designs but from action research carried out by the teachers. On the basis of before- and after-achievement scores, for each of the interventions, teachers identified what they judged to be greater than usual improvement in class scores for each of the measures. In the Technology class, 3 of the 24 students did not show improvement in their product evaluation scores. Although these scores were not gathered in an experimental study, they are scores assigned in the same way that regular class grades are determined. In each class, the improved level of performance was not limited to achievement test scores but was also observed in what might be seen as more general capabilities, such as knowledge about learning, critical thinking, and observations of learning process, such as the self-explanations during the Mathematics classes and the reflection procedures used in Teacher E’s class. Teacher E noted that, after the intervention, the students were more active during class:

“They are more likely to put up their hand and ask questions. They’re more likely to seek clarification; they’re more likely to expand on their workload. So not just doing the simple answers, they are checking in with me—‘Is this enough? What else do I need to do?’—so they are literally pushing themselves.”

The reports of the teachers suggested that the outcomes of each of the interventions yielded meaningful positive student outcomes of direct use for the teacher. Teacher C made the following reflection on his use of the ICAP framework:

“Gaining an understanding of the ICAP model helped me to adjust my teaching practices to integrate more Interactive lessons and foster a collaborative environment.”

**Table 2:** Effects of the teacher-designed class interventions (2021).

Year 8 Algebra (Teachers A and B)	Year 8 Technology (Teacher C)	Year 8 Civics and Citizenship (Teacher D)	Year 9 Humanities (Teacher E)
Measures Before and after intervention problem-solving quiz: problems ranged from simple to complex. Frequency of explicit strategy use by students. Scores on Knowledge/Understanding and Communication scores on IBYMP Criteria.	Measures Frequency of engagement in groups about product evaluation was initially difficult for students. Frequency of use of Interactive engagement processes.	Measures Text comprehension test score. Student reading strategy score. Student strategy use score. Thinking critically score.	Measures Strategy self-reports. Class grades. International Baccalaureate Middle Years Program (IMBYP) Criteria.
Outcomes Improvement in IBMYP Criteria grades Knowledge and Understanding (1-grade improvement on 4-point scale) Communication (1.5-grade improvement on 4-point scale). Increase in frequency of explication of strategies and frequency of explanations.	Outcomes Improvement in grades for 21/24 students compared to previous product evaluations.	Outcomes Improvements in text comprehension score. Improved learning strategy knowledge, strategy use, and thinking critically scores	Outcomes Positive changes in self-reports of strategy use. Higher scores on reflection scale. Student reports of how strategy use could be improved. More identification of what could be done better. Grade improvement from Term 2 to Term 3 for 43% of class. Higher scores for students on IBMYP criteria.

“While reflecting during the process, I realized I do a lot of implicit teaching and have worked to be more explicit when presenting strategies to students. I focused a lot on the evaluation and testing process with my students, and there was a clear improvement across the board [not only] with their grades but [also], more importantly, their engagement. Design Technology has always been an area which promoted self-regulated learners, but I feel the strategies I have learned and developed help me to better explicitly teach these strategies to more students and achieve more engagement going forward. I have even brought some of these strategies into classes with my senior students in 2023, and am noticing improvement in grades and engagement.”

In their reflection on the effects of the Mathematics class intervention, Teachers A and B noted the interaction of the SRL and ICAP frameworks:

“Training students to use the explicit strategies to explain to each other had a strong effect on ability to build knowledge and understanding. Using an interactive activity complemented the explicit practice well, and as a bonus, it seemed to increase engagement among less-motivated students.”

### 3.3. Results of Learning Sprints

For the remainder of this section, we will focus on the descriptions of a Learning Sprint provided by the teacher of a Biology class (Teacher F) during an interview. Teacher F worked with the Innovative Pedagogy Leader and other teachers to plan an intervention that focused on the use of a retrieval practice strategy. Retrieval practice is a strategy involving repeated recall of knowledge about a topic or concept and is sometimes referred to as self-testing or repeated retrieval. The act of recall of knowledge related to the topic is distinguished from the reviewing of notes that a student has made on that topic. So, when using this strategy, the student must recall the topic knowledge from memory without the assistance of the notes. This retrieval practice strategy has been shown to be very helpful for students at all levels of education, particularly after they have carried out some initial learning on a topic (Carpenter et al., 2022; Dunlosky & Rawson, 2019).

Teacher F had observed that her Biology students’ performance on examinations or tests was lower than on the more frequent portfolio tasks that students completed during regular class assessments, something that appeared to cause anxiety in some students. She indicated that her interest in use of the strategy arose:

“…because a lot of the students will do quite well in their written folio tasks, but when it came to their external assessments, such as an exam or their test, they would drop around a grade band or two grade bands from their folio tasks…a lot of students don’t pick science because it’s got tests. And so that’s, you know, that’s really sad that they don’t want to do something that they’re interested in just because they don’t think they’re good at tests…I think that it’s our responsibility to show them, you know, these ways of actually learning new concepts and doing it effectively.”

Prior to introduction of the retrieval practice strategy, Teacher F gathered performance data on assessment tasks that could be compared to that on similar tasks after the intervention. Her introduction of the strategy included information from research that indicated that the strategy had been shown to be effective and that it was more effective as a study strategy than just writing and reading notes.

“I explained to them …why we’re doing it is because when you do a test …you’re not going be able to look back at your notes. And it’s about training your brain to go back and remember the information that we did a while ago. And it will help you understand it better than just rewriting notes over and over again.”

By way of example, in a lesson, Teacher F would provide a prompt on the screen that might require students to write down all they knew about a cell membrane or cell structure. Then, she would give the students two minutes to review their notes on that topic so that the topic was not too daunting. Afterwards, books were closed, and the students then had around six minutes to recall their knowledge on the topic. After that, the students carried out an evaluation of their recall and reflected on potentially useful future learning actions to improve their knowledge. During the period of retrieval, students were advised to

“Write as much, mind map, draw, whatever they wanted, to get information on that particular topic into their books and it was writing by hand. And then, once the time was up, I’d give them another two minutes or three minutes with a different-colored pen to go over and check with their notes [to see] whether what they wrote was either right or wrong. And I’d get them to set a little goal at the end of it… maybe some things that you struggled with, what did you find challenging, what did you need to go to revise. And then, towards the end, I’ll get them to read each other’s books… to see what other people wrote and see if it was the same or different, to help them maybe set [their new] goal as well.”

Following her experience with retrieval practice, Teacher F observed that students wrote better notes and knew how to correct their work more effectively, with them perhaps noticing that a diagram was incorrect, or a concept not included in their recall, or labels for components of a diagram omitted. She observed that the requirement to examine other students’ work was helpful: “It helped when I was getting them to look at each other’s work because obviously other people’s retrieval would be different because I’m only giving them a small prompt to write about.” Some students also indicated that they were using the strategy independently in their study periods:

“A lot of them said—especially a lot of the students that were on that B level and they were trying really hard to get to that A—they said ‘I did some retrieval last night’ or, you know, ‘I did the practice questions, and then I did a bit of retrieval.’ So, it was starting to get them to use it in their own time.”

In the three surveys conducted by the LCG leaders across the year of the Learning Sprint program, retrieval practice was reported by Year 12 students to be the most beneficial for their learning. Use of the strategy during preparation for the Year 12 external examination was noted in an interview by the former LCG leader who had completed a year at university at the time of the interview:

“But I think [regarding] the stress and my confidence going into the exam, my confidence was increased, and my stress was decreased because that week, or two weeks prior to exams. I wasn’t cramming. I wasn’t sitting there rapidly trying to learn stuff. I had it already done. I was quite chill and calm, and just did a couple more of the strategies throughout that week.”

Teacher F also noted changes in her knowledge as a result of presentation of a Learning Sprint procedure. When asked to consider the explicit teaching of learning strategies in general terms, Teacher F contrasted the situation of herself as a student and that of her current students: “At university… I didn’t know how to learn. I really struggled… you know. I did Chemistry 1A, and it was so, so hard because I didn’t know how to learn it.”

### 3.4. Teacher Interventions, Learning Sprints, and the Use of Research Ideas

The data generated in the teacher intervention and Learning Sprint action–research programs and interviews can be related to two issues of research use discussed by Sato and Loewen (2022) and Mausethagen et al. (2025). The first of these concerns collaboration between researchers and teachers. Sato and Loewen (2022) identify one factor supporting greater use of research by teachers as a *collaborative mindset* between researchers and teachers, which they describe as “a mutual understanding of the facts that researchers and practitioners possess different types of professional knowledge yet share the common goal of promoting student learning” (p. 515). As noted earlier, in the case of the teacher interventions carried out by the teachers, both the researchers and teachers discussed the design of the interventions and the details of the results noted by teachers at the completion of the interventions. These discussions included consideration of how specific research ideas could be included in lessons. In addition, the leaders and teachers continued to involve the researchers in discussions of ways to present information to their fellow teachers. We see that these instances of collaboration were likely to have increased the likelihood of the use of research ideas presented in the PDP.

Another obstacle identified by Sato and Loewen (2022) as impacting on the use of research ideas concerns time, particularly the time involved in enacting a strategy intervention in class lessons. This obstacle to use of research has been observed in research such as that by Nibali (2017), where teachers explained their subsequent lack of use of a period of training in promotion of SRL strategies in their teaching as resulting from the extra time such teaching would add to their lessons. In their explanations for this lack of use of professional learning and SRL strategy promotions, these teachers emphasized the pressure they experienced to get through curriculum content listed in the official syllabus. By way of contrast, after the Learning Sprint program, Teacher F remarked on the amount of time taken to teach the strategies during lessons:

“When I am doing a spaced practice, they are learning about the curriculum, or when they are doing a retrieval, they are still learning the curriculum. So, I don’t see those as separate, because when they’re completing that learning strategy, it’s still curriculum.”

This consideration of time associated with strategy promotion also emerged in other interviews. Teacher E made a similar argument with respect to the time taken for the strategy instruction during her intervention: “You just embed it within the task, which is no extra time.” Teacher A made a distinction between the time effects of attending to learning strategies during lesson preparation and in class teaching: “But I think, really, it was more the work that the teachers had to do to think about how they were constructing their learning activities more than the time [taken] in the classroom.”

However, it is also relevant to note that, with respect to this concern about strategy promotion in lessons taking up lesson time, these teachers (and others involved in the PDP) did remark on the significant, and possibly deleterious, pressure that the current design of the Australian curriculum placed on teachers to “get through” the curriculum. In his reflection on the school’s actions, Leader 2 noted that

“I think the culture of our teachers towards their own practice has changed. And now, it’s acceptable for, or it’s an accepted practice, for us to complete small projects like that, and so our teachers are more reflective; our teachers see themselves more as highly effective practitioners. I think that the overall impact at the whole-site level is getting that credibility, the evidence-backed strategies—bringing them in—and then the teachers actually trialing them within their own context.”

### 3.5. Results of the Learning Culture Group

The LCG could be seen as a part of the program of activities carried out within the school that systematically made provision for furthering the opportunities for students to support their own growth in knowledge about learning and the knowledge growth about learning of other students. This is highlighted here as an uncommon instance of the reporting of student promotion of knowledge related to SRL to other students. In the words of Student 1, the LCG was designed to involve

“…students who really care about the learning experience at our school…So, over a number of years, as we joined the group, we started hearing about learning strategies, and the Learning Scientist is one organization, one group of experts, who have developed some strategies and that really inspired us to really take it further across the whole school… to say these are strategies we want to share with others—they work really well. They’re evidence-based. And so, it’s been this very progressive thing we’ve worked up from having a vague idea if we want to improve learning to then saying ‘OK, here’s some concrete ways we can actually improve learning.’ We can promote these strategies; we can teach other students about what we know.”

The group evolved a regular meeting schedule that also involved teachers and a team-based approach using online interactions on Microsoft Teams and seeking out learning strategy resources, such as those presented on the Learning Scientists website (https://learningscientists.org). In terms of having an impact on students’ use of learning strategies, it was seen that the LCG could provide a source of encouragement of such use to the wider student body, additional to that which was provided by teachers. As noted earlier, the LCG students took up the ICAP framework after hearing that it had been discussed in the PDP and used it in their presentation to teachers and researchers. In 2022, it was the student leader of the LCG who introduced the idea of Learning Sprints to Year 12 students at a school assembly. During that year, as the Learning Sprints were running, the LCG leaders also surveyed all Year 12 students three times about their views on the learning strategies being promoted and students’ use of them.

Another one of the activities of the LCG has been to give advice to students who attend its regular meetings in order to ask questions about their own learning and inform them about how they could change their learning practices.

“We’ve had a lot of Year 9s come in, and they aren’t the most learning-focused people. Yet, you can see, over time, they change their goals a lot…And they’ve been bringing people in as well. I think, when we started, we had a few Year 9s come in. And then, a few weeks later, like all their friends were there. They were telling other people; they were informing them. We were getting them in on that practice. And I think that’s really important” (Student 2).

It is also relevant to note that the LCG activities, such as their involvement with teachers during the student-free days that focused on the details and effects of the 2021 learning strategy interventions by teachers, also had the potential to influence other teachers’ views about learning strategies. One specific effect reported by Student 1 was the increase in explicit discussion of learning strategies in class:

“A lot of teachers are really starting to be explicit about it. It helps us as students to identify strategies that we can then later use in our own independent study. But it also makes it a lot clearer to students that there is a framework being used in a process from the teachers.”

### 3.6. Student Approaches to SRL and Its Promotion

A notable feature of the interviews with the LCG leaders was their knowledge about self-regulated learning and methods of its promotion in class teaching. Much of this discussion could be seen as similar to that which might occur in a research team meeting. Examples of the students’ reports on these topics are included in Table 3. Across these reports, we see conceptions of learning and its management that are quite consistent with the accounts of SRL in contemporary literature (e.g., Dunlosky & Rawson, 2019; Zimmerman, 2013). Learning is sometimes assumed to be something that is natural, that we automatically know how to do effectively as humans. By way of contrast, these students see that they have learned additional, new strategies that can have major benefit for their classroom learning. These reports also make explicit the shared responsibility for learning that needs to be exercised by teacher and student, supporting the reminder given by researchers such as Biggs (2012), Shuell (1988), and Simon (1998) that the teacher can exert a limited influence on the outcome of any learning episode, with the ultimate effect also being necessarily dependent on the learning actions used by the student.

### 3.7. Student Assessments of Learning Culture Group Activities

In a final discussion of the learning strategies undertaken by the school, two of the student leaders of the LCG were asked to reflect on the effects of the learning strategies in which their teachers and themselves had been involved. Student 3 stated,

“Learning about and sharing with teachers Interactive, Constructive, Active, Passive learning in Year 9 helped me identify the spectrum of activities being used in the classroom. I enjoyed demonstrating and promoting positive examples of interactive and constructive activities on student-free days. I have noticed the impact of this in some of my classes, such as Modern History and English Literary Studies, where more interactive/constructive activities have been prominent in my later years at school. For example, answering comprehension questions or assessment-style practice questions related to a topic or concept…

It’s uncommon for secondary students to confidently discuss pedagogy, let alone understand it, but the Learning Cultures students could.”

This student reported that their experience in the school program of use of the PDP research ideas meant that they came to know these ideas well enough that they become second nature and a core part of learning and independent study. For them, learning about ineffective and effective learning strategies had been very impactful, especially for content-heavy South Australian Certificate of Education Stage 2 subjects like Chemistry and Biology.

For Student 3, once the retrieval practice strategy was introduced, she used this strategy in her own independent study on a daily basis, especially when assessments were approaching. She reported that the most important thing she learned at Blackwood High School was learning how to learn. She reflected that

“…having a toolkit of strategies that I have used frequently, I know that if I struggle learning new concepts at University, I feel confident that I can learn anything with these strategies to fall back on. I know that if one strategy doesn’t work for me on a specific task, I have more to try. Having practiced them since Year 9, I also feel confident that they actually work. I know from personal experience that they live up to the evidence that they are based on in scientific studies.”

In their joint discussions, these students reported that frameworks such as the ICAP framework and SRL strategies facilitated more effective exam and in-class assessment preparation for skill-based subjects, for example, where deep analysis was required. Their experience has been that when teachers were explicit about such strategies, supported by posters for students made by the LCG, students were more successful in employing them: “We recommend other schools to be mindful of this when implementing these approaches.”

In his reflection on his future studies, Student 1 noted that

“I have chosen a career in education partially as a result of my experiences in learning about learning, metacognition, and pedagogy throughout secondary school… I have been inspired by the work that goes around innovation in education and wish to play my part in the future as an educator. In terms of further tertiary study, I’m sure that the strategies and devices that we’ve explored will prove beneficial.”

### 3.8. Teachers’ Views of the Learning Culture Group

For the school leaders, one of the unanticipated effects of the involvement of both teachers and students in explicit discussions about learning was the realization that poorly developed knowledge about learning in students was also likely to be associated with a lack of student knowledge about the nature of teaching. As noted by Leader 1,

“I’ve even had students comment …’We just thought that you pulled the book off the shelf and kind of taught from that.’ Because I guess the notion of a textbook is there, and so surely there’s an accompanying book that says this is the teacher’s version, that you just teach it. And I said, ‘well, no.’ And when we got students involved with our student-free days, and they actually came in and observed us, you know, through our Learning Cultures Group, and they observed and worked on alongside us. And their initial reflections were one of, ‘We had no idea teachers put so much thought and planning and time into the preparation of the lessons that you deliver.’”

Another effect noted by this school leader was that the involvement with students in discussions of learning could be seen as a type of “collaborative professionalism” in which knowledge of teaching and learning was made more public for students, as indicated in the example immediately above. In his view, this sharing of knowledge about learning strategies was a driver of the development of student agency discussed in educational policy and educational research. Making learning strategies public, with both teachers and students involved in the public discussion of the strategies, was seen to change the learning environment in classes so that there was not “such a heavy reliance on the teachers to be the drivers of learning” (Leader 1). The argument made by the LCG leaders provided support for this teacher’s view:

“It helps us as students to identify strategies that we can then later use in our own independent study. But it also makes it a lot clearer to students that there is a framework being used in a process from the teachers.”

### 3.9. Students’ Use of Research Ideas

The actions and the interview responses of the students show evidence of their use of research ideas presented to their teachers in the PDP and of other SRL research used by those teachers. As noted above, we observed the students using the ICAP framework in a presentation to teachers and researchers and also in subsequent workshop presentations they made to their fellow students. In the interview responses, we see that they have focused on research ideas related to SRL, to explicit promotion of strategies, to the use and value of specific strategies, and to the key role of the student in learning, as described by Simon (1998). In these instances, we see that key messages to be derived from SRL research findings have been understood and used by these students in their classroom lessons and private study.

### 3.10. Leaders’ Reflections on the Use of Research Ideas in the School

In their interview reflections on the involvement of the teachers and students at this school, the two school leaders identified several important features of the period following the end of the PDP in 2020. They noted that themes emphasized in the PDP research project reinforced their experiences in the school where they both taught prior to coming to their current school. A component of the PDP that was of particular significance for them was the need for explicit promotion of SRL strategies. For Leader 2,

“Explicit promotion is a key… I think it opened my eyes around what students are actually taking in rather than what teachers are saying. Because teachers assume that because they’ve done something over and over again that the students know why they’re doing it, and they know what that strategy is actually achieving. But really, sometimes students, I think most students, don’t really, they’re just following along.”

Leader 1 drew attention to the fact that participation in the PDP highlighted, for him, the need to strengthen the teachers’ knowledge about learning:

“And whilst they are discipline experts and they are pedagogical experts, they may not be learning experts, because that’s again a bit of a niche area in terms of education: How do we support students to be better learners but [also] how do we actually explicitly instruct on how to learn? And that was that kind of piece of the puzzle, I think, that we were looking at trying to find and put it in place.”

This comment also draws attention to the surprising situation in which knowledge about learning is seen to be a “niche area.” In all teaching, students are required to learn. But in some of this teaching, the ‘how’ of the learning is not given sufficient attention. A related point made in interviews was that the emphasis given to the need for explicit teaching of the strategies also served to remind teachers of the need in their lesson planning to consider not just what to teach, but also to consider how to teach that content, and also how the content might be learned. We see in previous comments from both teachers and students that use of the ICAP framework reinforced this focus on the ‘how’ for teachers, as they considered the types of tasks they could include in lessons that would stimulate Constructive and Interactive levels of cognitive engagement.

In their analysis of the issue of teachers’ use of research, Joram et al. (2020) argued that within-school support can increase use of research findings. In the current study, we see examples of such support in two areas of collaboration that developed within the school’s extension of the findings from the PDP. The first of these was collaboration among teachers as they worked at helping one another to introduce strategies in the Learning Sprints. For this, teachers within curriculum areas helped one another organize key components of the curriculum content so that they could then consider together procedures for teaching the SRL strategies. This latter activity was seen to give the teachers increased access to a common language about learning processes, a language that was specifically focused on how to teach the content. This collaboration among teachers can also be seen to have made on impact on levels of teacher agency for use of the research ideas, a factor seen as relevant in Mausethagen et al.’s (2025) analysis of research use.

The second area of increased collaboration was the continuing interaction between teacher and students in paying explicit attention to learning strategies and processes of learning. This emerged in comments like the one from earlier about the need to improve students’ knowledge of study techniques, and when it was noted that the discussion of strategies with students brought increased attention to the point that teachers could not be the only ‘drivers’ of student learning. As is evident in the section on ‘responsibility for learning’ in Table 3, the relevance of this point for students was interpreted in a similar way by student members of the LCG.

A final point of reflection by leaders was the positive impact of the school project on student achievement. This was evident in the reports on effects of the teacher interventions in 2021 and after the Learning Sprints in 2022. Improvement in the levels of achievement were noted in the results of the external examinations in 2022 and 2023. The results achieved by students in SACE Stage 2 external examinations were higher in terms of aggregate A and B grades than in the previous five years.

## 4. Conclusions

This project began with the decision of leaders and teachers to accept an invitation to participate in a PDP presented as part of a research project. We see that the actions taken by the leaders, teachers, and students in this school in the years following the end of the PDP provide a clear example of ideas from research being used effectively to make a substantial impact on the learning environment and the teaching and learning practices in the school (Williams & Coles, 2007). As noted in the discussions of the teacher interventions and Learning Sprints, research ideas central to the PDP were used by teachers to modify their teaching practices. However, the account of this impact presented here is not an account associated with an experimental study of change; rather, it is an account of a systematic and innovative application of research ideas that involved gathering of data that could be examined critically. The classroom interventions in 2021 and 2022 were carefully planned and systematically executed. These interventions included the use of soundly based measures of achievement and strategy use that provided the basis for teachers’ judgements of meaningful change in students’ learning behaviors and a major change in teachers’ explicit promotion of learning strategies that has been a key educational research issue for some time (e.g., Dignath & Veenman, 2021; Vosniadou et al., 2024). As noted above, the project was also associated with beneficial impacts on students’ external examination performance. The influence of ICAP is evident in the comments of both the teachers and the students and in the teaching provided to teachers and researchers in their presentation on the student-free day. It is clear that the use of learning strategies has taken place in a context that supported an interactive collaborative culture and the use of constructive activities. This suggests that the PDP’s emphasis on the promotion of learning strategies in the context of a learning-how-to-learn culture had been taken up by many teachers and students in this school.

In considering the possible influences on the use of research ideas by teachers and students in this school and their implications for other researchers and schools, two broad features of the whole research–school use event have been noted. The first of these relates to the close connection between the PDP and the teaching practices of the teachers participating in the research. In terms of the obstacles to research use noted by Sato and Loewen (2022), the PDP brought the research ideas and the participants’ own teaching actions into joint focus. This might suggest to other researchers that use of research ideas by teachers is more likely to be facilitated when the teachers use those ideas in examination of their own teaching practices.

The second argument made above is that the collaborative discussions between researchers and teachers, among teachers, and between teachers and students related to the research ideas can be seen to have encouraged use of the research ideas presented in the PDP. These discussions could also be seen as being likely to increase the levels of agency of both the teachers and the LCG students with respect to the research ideas and their use in teaching and learning. In recent research by Joram et al. (2020), Mausethagen et al. (2025), and Sato and Loewen (2022), these practices were portrayed as likely to increase the use of research ideas in schools. A related perspective on the effects on research discussed here is identified by Rust (2009) and Williams and Coles (2007); Rust refers to as “border crossing”. The situation that developed in this research–school use of research enabled both the researchers and teachers to see the relevance of the actions being undertaken by both groups, meaning that possible borders between the different worlds of the researchers and teachers were not highlighted. Both groups could see the activities undertaken by the teachers and students as flowing clearly from the research. In this context, the level of agency of the teachers and the students was also likely to have been increased (Joram et al., 2020). Other researchers may explore whether greater uptake of research ideas by teachers can be facilitated by such border crossing.

Of particular note in this school has been the students’ innovative patterns of involvement in both the planning and progression of the school initiatives on knowledge and promotion of teaching and learning strategies. The activities of students in explicit promotion of learning strategies observed by the research team and the accounts of students in the interviews provide evidence of a level of quite sophisticated knowledge and use of research ideas that is uncommon. Prior to the research beginning, students in this school had been collaborating with leaders and teachers in promoting learning strategy information that has impacted teachers and other students. In terms of educational research, these actions provide a very useful model for showing how promotion of knowledge about learning can be enacted by students, for themselves and for other students, at the necessary site—in the minds of the students who must do the learning. Simon (1998) put this perspective quite bluntly: “Learning takes place in the minds of students and nowhere else, and the effectiveness of teachers lies in what they can induce students to do.” (p. 346).

It might be seen that there could also be implications, stemming from the actions of this school, for policy adopted either by individual schools or education departments. At the individual school level, the approaches adopted by the school discussed in this research might prompt discussions with researchers about how planned research projects involving a school could be carried out. One such possibility is that school leaders might consider whether they invite researchers to set up border-crossing procedures for the planned research so that use of research ideas shown to be effective in teaching could be incorporated more readily into the research design. Similar considerations might be considered at the level of an education department that is asked to incorporate research into its schools.

## 5. Limitations

As noted at several points in this paper, the data and results presented herein did not arise from an experimental design, and so they must be interpreted with caution. Nevertheless, the findings observed in relation to teacher and student practices related to the research ideas included in the PDP in this school can be seen to suggest that the methods used in the PDP and in the interactions between researchers and teachers provide an encouraging basis for further systematic investigation of issues of both research use and student and teacher promotion of SRL strategies. The characteristics of the PDP, identified as addressing obstacles to research use, could be made the subject of experimental investigations related to effective use of research ideas by teachers. In addition, the existence of the student Learning Culture Group, and its activities noted in this paper, could also stimulate exploration of an important, and possibly underused, avenue for stimulation of the growth of student knowledge of learning within the classroom.

## Figures and Tables

**Table 3:** Examples of students’ views of self-regulated learning and its promotion during interviews.

Elements of SRL and Its Promotion	Student Reports
Learning environment	Over a number of years, as we joined the group, we started hearing about learning strategies… it’s been this very progressive thing we’ve worked up from having a vague idea if we want to improve learning to then saying ‘OK, here’s some concrete ways we can actually improve learning.’ We can promote these strategies; we can teach other students about what we know. There might be a whole-school approach to learning improvement that they want to bring into the group and get some student insight into (Student 1).
Self-regulated learning	I think self-regulated learning is a learner being able to identify appropriate strategies to use in their learning. It’s about identifying challenges, identifying what a learner can do to really, to learn the content…I think that doesn’t come naturally to a lot of people. It’s built up over time, and going back to learning strategies again, that’s been a big focus of ours. It’s about students developing their way of using those strategies, so, people don’t just walk into high school and go, ‘right, I know how you do retrieval practice or spaced practice.’ It’s our role to promote those strategies, to teach them how to use it, but also the teachers role as well (Student 1).”
The ‘self in self-regulated learning	I feel like self is very much emphasized there because it’s something we want people to be doing independently… OK, yes, I can learn about it and harness it here, but this is also something I need to improve personally, because this is something that I’m doing for myself and it’s something to do for myself. I think being a self-regulated learner is to recognize that you need to do your bit as well in the classroom and outside of the classroom (Student 3).
Responsibility for learning	I think there’s definitely a shared responsibility…At the beginning of a concept, a teacher might take on a significant amount of the responsibility and the learning by going through content, asking questions, answering questions rather. But over time, I think it’s really important that students assume more responsibility for their learning… To go back to your question, the teacher has some responsibility in the learning, but again, I think the student, the learner, is definitely also part of that equation of responsibility (Student 1).
Teacher modeling of strategies	I think the big thing is rather than just saying this is the strategy, this is how we do it…it’s really important to model it and actually have students work through that…But when they actually see how effective it can be or they realize it might be a better way rather than cramming or some other methods they’re using in the independent study…That prompts students to use the strategies in their own time. If [students] can’t see how it applies to them personally, they’re probably not going to retain it…So modeling it, especially in that way will help them retain it (Student 1).
Retrieval practice effect on learning	You’ve got two ideas, and you’ve connected them. So, you come back a week later, and you retrieve this connection. Why does…coming back to it help you with that connection? Makes the connection stronger, I think…If you remember this one, the link is still there to like… draw on it quicker (Student 4).
Metacognitive monitoring	I think the process of saying if it’s correct or incorrect is that other process of ‘OK yeah, I’ve got it or no, I haven’t got it, but this is why I missed it’ (Student 4).
The brain	The teacher is teaching you some concept… And the first time you meet it, it’s like this. Well, when you’ve done some of that retrieval practice, what happens to this representation? It broadens out so it becomes like a mind map. So that one topic is now either connected with other topics or it branches off into …like subtopics. I feel like a memory isn’t just of one thing, like it’s all interweaving. It’s like a, it’s a spider web, I think. Like our memory is connected in all channels (Student 4).
Areas for future attention	Teachers can also teach students how to do it in their own time as well, because that’s something I haven’t really seen. Yes, we can do it in lessons with the teachers… But how do we also do them in our own time (Student 2).

## Data Availability

The original contributions of this study are included in the article. Further inquiries can be directed to the corresponding author.

## Abbreviations

The following abbreviations are used in this manuscript:

IBMYP	International Baccalaureate Middle Years Program
ICAP	Interactive, constructive, active, passive
ICSEA	Index of Community Socio-Educational Advantage
LCG	Learning culture group
PDP	Professional development project
SACE	South Australian certificate of education
SRL	Self-regulated learning

## References

[B1-behavsci-15-01644] Biggs J. B. (2012). What the student does: Teaching for enhanced learning. Higher Education Research & Development.

[B2-behavsci-15-01644] Bjork R. A., Dunlosky J., Kornell N. (2013). Self-regulated learning: Beliefs, techniques, and illusions. Annual Review of Psychology.

[B3-behavsci-15-01644] Boekaerts M. (1997). Self-regulated learning: A new concept embraced by researchers, policy makers, educators, teachers, and students. Learning and Instruction.

[B4-behavsci-15-01644] Buchmann M. (1984). The use of research knowledge in teacher education and teaching. American Journal of Education.

[B5-behavsci-15-01644] Carpenter S. K., Pan S. C., Butler A. C. (2022). The science of effective learning with spacing and retrieval practice. Nature Reviews Psychology.

[B6-behavsci-15-01644] Chi M. T. H., Boucher N. S., Overson C. E., Hakala C. M., Kordonowy L. L., Benassi V. A. (2023). Applying the ICAP framework to improve classroom learning. In their own words: What scholars want you to know about why and how to apply the science of learning in your academic setting.

[B7-behavsci-15-01644] Chi M. T. H., Wylie R. (2014). The ICAP framework: Linking cognitive engagement to active learning outcomes. Educational Psychologist.

[B8-behavsci-15-01644] Cohen L., Manion L., Morrison K. (2017). Action research. Research methods in education.

[B9-behavsci-15-01644] Department for Education, South Australia (n.d.). Strategy for public education.

[B10-behavsci-15-01644] Dignath C., Mevarech Z. (2021). Introduction to special issue mind the gap between research and practice in the area of teachers’ support of metacognition and SRL. Metacognition and Learning.

[B11-behavsci-15-01644] Dignath C., Veenman M. V. J. (2021). The role of direct strategy instruction and indirect activation of self-regulated learning—Evidence from classroom observation studies. Educational Psychology Review.

[B12-behavsci-15-01644] Dunlosky J., Rawson K. A. (2019). The Cambridge handbook of cognition and education.

[B13-behavsci-15-01644] Dunlosky J., Rawson K. A., Marsh E. J., Nathan M. J., Willingham D. T. (2013). Improving students’ learning with effective learning techniques: Promising directions from cognitive and educational psychology. Psychological Science in the Public Interest.

[B14-behavsci-15-01644] Efklides A. (2017). Affect, epistemic emotions, metacognition, and self-regulated learning. Teachers College Record.

[B15-behavsci-15-01644] Fong C. J., Patall E. A., Snyder K. E., Hoff M. A., Jones S. J., Zuniga-Ortega R. E. (2023). Academic underachievement and self-conceptual, motivational, and self-regulatory factors: A meta-analytic review of 80 Years of research. Educational Research Review.

[B16-behavsci-15-01644] Gleeson J., Harris J., Cutler B., Rosser B., Walsh L., Rickinson M., Salisbury M., Cirkony C. (2024). School educators’ use of research: Findings from two large-scale Australian studies. Research Papers in Education.

[B17-behavsci-15-01644] Griffin G. A., Barnes S. (1986). Using research findings to change school and classroom practices: Results of an experimental study. American Educational Research Journal.

[B18-behavsci-15-01644] Gurung R. A. R., Dunlosky J. (2023). Study like a champ.

[B19-behavsci-15-01644] Hattie J. (2012). Visible learning for teachers: Maximizing impact on learning.

[B20-behavsci-15-01644] Hemsley-Brown J. V., Sharp C. (2004). The use of research to improve professional practice: A systematic review of the literature. Oxford Review of Education.

[B21-behavsci-15-01644] Huang C. T., Yang S. C. (2015). Effects of online reciprocal teaching on reading strategies, comprehension, self-efficacy, and motivation. Journal of Educational Computing Research.

[B22-behavsci-15-01644] Joram E., Gabriele A. J., Walton K. (2020). What influences teachers’ “buy-in” of research? Teachers’ beliefs about the applicability of educational research to their practice. Teaching and Teacher Education.

[B23-behavsci-15-01644] Kaberman Z., Dori Y. J. (2009). Metacognition in chemical education: Question posing in the case-based computerized learning environment. Instructional Science.

[B24-behavsci-15-01644] Learning Scientists (2025). https://www.learningscientists.org/.

[B25-behavsci-15-01644] MacArthur C. A., Harris K. R., Graham S., Urdan T., Bus A. G., Major S., Swanson H. L. (2012). Strategies instruction. APA educational psychology handbook, Vol. 3. Application to learning and teaching.

[B26-behavsci-15-01644] Mausethagen S., Hermansen H., Afdal H., Lorentzen M., Holmeide H. (2025). A systematic critical review of research on ‘research use’ in education: Towards more profession-sensitive conceptualisations. Educational Research Review.

[B27-behavsci-15-01644] Mayer R. E. (2017). Educational psychology’s past and future contributions to the science of learning, science of instruction, and science of assessment. Journal of Educational Psychology.

[B28-behavsci-15-01644] Nibali N. (2017). Teaching self-regulated learning: Teacher perspective on the opportunities and challenges. Annual Conference of the Australian Association for Research in Education.

[B29-behavsci-15-01644] Penuel W. R., Briggs D. C., Davidson K. L., Herlihy C., Sherer D., Hill H. C., Allen A. R. (2017). How school and district leaders access, perceive, and use research. AERA Open.

[B30-behavsci-15-01644] Pintrich P. R. (1999). The role of motivation in promoting and sustaining self-regulated learning. International Journal of Educational Research.

[B31-behavsci-15-01644] Queensland Academy for Science, Mathematics and Technology (2021). IB middle years assessment guidance handbook.

[B32-behavsci-15-01644] Renkl A., Eitel A., Dunlosky J., Rawson K. A. (2019). Self-explaining: Learning about principles and their application. The Cambridge handbook of cognition and education.

[B33-behavsci-15-01644] Rust F. C. (2009). Teacher research and the problem of practice. Teachers College Record.

[B34-behavsci-15-01644] Sato M., Loewen S. (2022). The research–practice dialogue in second language learning and teaching: Past, present, and future. The Modern Language Journal.

[B35-behavsci-15-01644] Schunk D. A., Greene J. A. (2018). Handbook of self-regulation of learning and performance.

[B36-behavsci-15-01644] Schunk D. H., Zimmerman B. J., Reynolds W. M., Miller G. E., Weiner I. B. (2013). Self-regulation and learning. Handbook of psychology: Educational psychology.

[B37-behavsci-15-01644] Shuell T. J. (1988). The role of the student in learning from instruction. Contemporary Educational Psychology.

[B38-behavsci-15-01644] Simon H. A. (1998). What we know about learning. Journal of Engineering Education.

[B39-behavsci-15-01644] Sitzman T., Ely K. (2011). A meta-analysis of self-regulated learning in work-related training and educational attainment: What we know and where we need to go. Psychological Bulletin.

[B40-behavsci-15-01644] Usher E. L., Schunk D. H., Schunk D. H., Greene J. A. (2018). Social cognitive theoretical perspective of self-regulation. Handbook of self-regulation of learning and performance.

[B41-behavsci-15-01644] Vosniadou S., Bodner E., Stephenson H., Jeffries D., Lawson M. J., Darmawan I. N., Kang S., Graham L., Dignath C. (2024). The promotion of self-regulated learning in the classroom: A theoretical framework and an observation study. Metacognition and Learning.

[B42-behavsci-15-01644] Vosniadou S., Lawson M. J., Bodner E., Stephenson H., Jeffries D., Ngurah Darmawan I. G. (2023). Using an extended ICAP-based coding guide as a framework for the analysis of classroom observations. Teaching and Teacher Education.

[B43-behavsci-15-01644] Vosniadou S., Stephenson H., Lawson M. J., Jeffries D. (2025). A professional development program that combines direct with indirect promotion of self-regulated learning for secondary school teachers. Behavioral Sciences.

[B44-behavsci-15-01644] Williams D., Coles L. (2007). Teachers’ approaches to finding and using research evidence: An information literacy perspective. Educational Research.

[B45-behavsci-15-01644] Willingham D. T. (2023). Outsmart your brain.

[B47-behavsci-15-01644] Winne P. (2018). Theorizing and researching levels of processing in self-regulated learning. British Journal of Educational Psychology.

[B46-behavsci-15-01644] Winne P. H., Zimmerman B. J., Schunk D. H. (2001). Self-regulated learning viewed from models of information processing. Self-regulated learning and academic achievement: Theoretical perspectives.

[B48-behavsci-15-01644] Zimmerman B. J. (2013). From cognitive modeling to self-regulation: A social cognitive career path. Educational Psychologist.

